# Digital Health Intervention for Asthma: Patient-Reported Value and Usability

**DOI:** 10.2196/mhealth.7362

**Published:** 2018-06-04

**Authors:** Rajan Merchant, Rubina Inamdar, Kelly Henderson, Meredith Barrett, Jason G Su, Jesika Riley, David Van Sickle, David Stempel

**Affiliations:** ^1^ Dignity Health Woodland Clinic Medical Group Woodland, CA United States; ^2^ Dignity Health Mercy Medical Group Sacramento, CA United States; ^3^ Propeller Health Research and Development San Francisco, CA United States; ^4^ University of California Berkeley Berkeley, CA United States; ^5^ Propeller Health Research and Development Madison, WI United States

**Keywords:** asthma, mHealth, surveys and questionnaires, patient satisfaction, perception, self-management

## Abstract

**Background:**

Although digital health tools are increasingly recognized as effective in improving clinical outcomes such as asthma control and medication adherence, few studies have assessed patient experiences and perception of value.

**Objective:**

The aim of this study was to evaluate patient satisfaction, perception of usability and value, and desire to continue after 12 months of using a digital health intervention to support asthma management.

**Methods:**

Participants were enrolled in a randomized controlled study evaluating the impact of a digital health platform for asthma management. Participants used electronic inhaler sensors to track medication use and accessed their information in a digital health platform. Electronic surveys were administered to intervention arm participants aged 12 years and older after 12 months of use. The survey assessed asthma control, patient satisfaction with the sensor device, and perception of the usability and value of the digital health platform through closed-ended and open-ended questions. Logistic regression models were used to assess the impact of participants’ characteristics on survey completion, satisfaction, and perception of value.

**Results:**

Of the 207 intervention arm participants aged 12 years and older, 89 submitted survey responses (42.9% response rate). Of these 89 participants, 70 reported being very satisfied (79%, 70/89) or somewhat satisfied (20%, 18/89) with the inhaler sensor device. Moreover, 93% (83/89) expressed satisfaction with the reports, and 90% (80/89) found the information from the reports useful for learning about their asthma. In addition, 72% (64/89) of the participants reported that they were interested in continuing to use the sensor and platform beyond the study. There were no significant differences in satisfaction with the device or the platform across participants’ characteristics, including device type, age, sex, insurance type, asthma control, or syncing history; however, participants with smartphones and longer participation were more likely to take the survey.

**Conclusions:**

Electronic sensors and a digital health platform were well received by participants who reported satisfaction and perceived value. These results were consistent across multiple participants’ characteristics. These findings can add to a limited literature to keep improving digital health interventions and ensure the meaningful and enduring impact on patient outcomes.

## Introduction

### Cost of Asthma

The health impact and economic costs of asthma are significant, with the annual direct costs approaching US $50.1 billion and indirect costs such as lost productivity contributing an additional US $5.9 billion [1-3]. Despite asthma’s negative impact on health, patient self-management remains a challenge, with controller medication adherence rates of approximately 30% [4-7]. Asthma self-management requires daily work by the patient, including adherence to complex medication regimens and addressing multiple triggers of symptoms.

### Digital Health Interventions for Asthma

Digital health interventions have been used increasingly in self-management interventions for asthma. For example, the development of mobile apps for asthma management doubled between 2011 and 2013 [8]. These interventions, which can include tools such as short message service, mobile apps, Web-based portals and websites, and electronic inhaler sensors, offer new ways for patients to monitor and manage their asthma. A limited but growing body of literature demonstrates the effectiveness of digital interventions in improving clinical outcomes, including asthma control, adherence, and symptom-free days [9-24].

Better understanding of how patients perceive and use digital health interventions to achieve improved outcomes is of utmost importance; however, there have only been a few studies published that focused on this topic. A recent review of digital health interventions across multiple diseases found that usability was assessed in only 33% of studies [25]. Within the asthma literature, a systematic review concluded that “patient perspectives have been largely ignored” [26]. A small number of studies have evaluated patient perceptions of asthma apps [19,24] or electronic inhaler sensors [27,28], but these have been limited to small samples over short periods of time (1-4 months). Little is known about how patients perceive the utility of combining electronic inhaler sensors with mobile apps and other digital tools. Furthermore, no studies have explored patient perceptions in a real-world setting over a sustained duration.

The study’s primary objective was to evaluate participant satisfaction, perception of usability and value, and overall experience after sustained use of a digital health intervention combining sensors, a mobile app, a Web dashboard, and email communication, to support asthma management in a real-world setting. The study also aimed to explore whether these perceptions would be influenced by specific participants’ characteristics such as age, gender, device ownership, insurance type, asthma control, and engagement. Improving the understanding of how diverse patients experience digital tools for asthma self-management will contribute to the success of these tools and the durability of their effects.

## Methods

### Digital Health Platform

**Figure 1:**
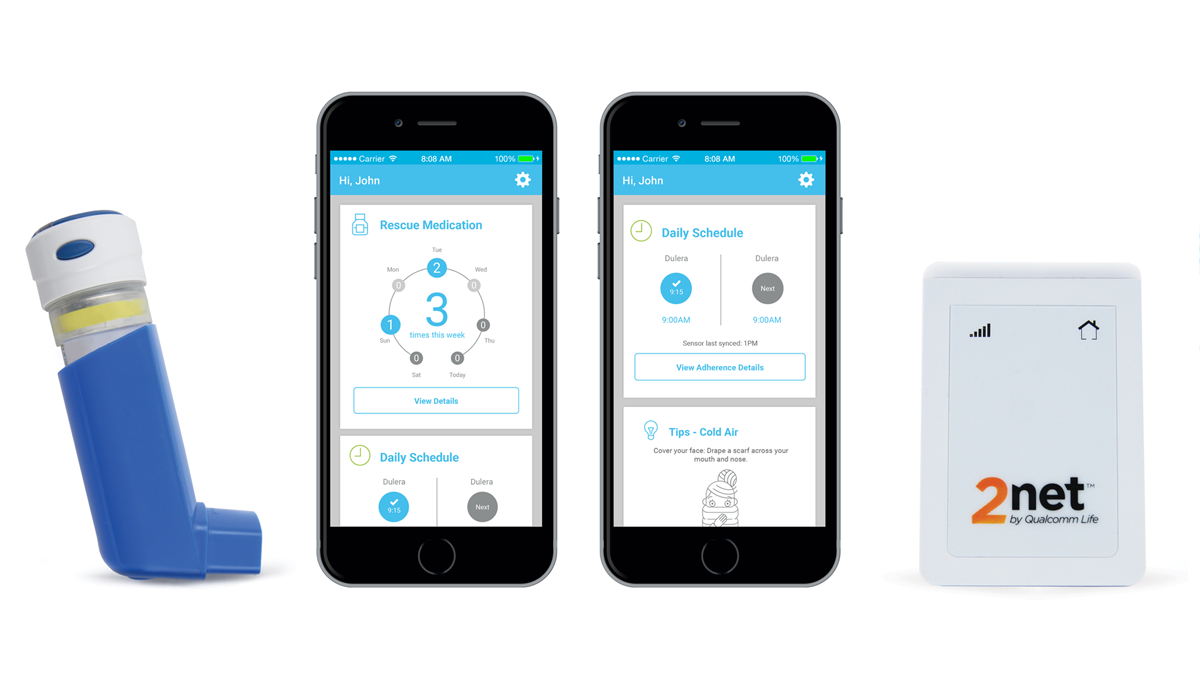
Propeller Health sensor device, smartphone app, and hub base station. The Propeller sensor attaches directly to the metered dose inhaler and objectively captures the date, time, and frequency of medication use. The sensor transmits these data wirelessly via Bluetooth to a paired smartphone, where a mobile app displays the information for the user. For participants without a smartphone, a wireless hub transmits the data, which are accessible through a Web-based dashboard.

Participants were enrolled in a randomized controlled study to evaluate the impact of a Food and Drug Administration–cleared digital health platform on asthma outcomes including short-acting beta-agonist (SABA) use and asthma control. The digital health platform includes electronic inhaler sensors (Propeller Health, Madison, Wisconsin) that attach to inhaled asthma medications (Figure 1). The sensor monitors the date, time, and frequency of medication use and transmits these data back to secure servers through a smartphone app or hub base station. Location data are collected on medication use among patients who have a smartphone. The sensors regularly transmit data back to the server, or sync, through the smartphone or hub. The version of the sensor that participants used in this study required them to charge their sensors every 2 weeks.

The data collected by the sensors are presented back to patients and health care providers through the digital health platform. The platform aims to promote disease awareness and self-management by enabling access to a patient’s own medication use data, including daily assessments of asthma control, adherence, identified triggers, and education based on the national guidelines (NHLBI 2007). The information is shared via a number of communication channels for all users, including weekly email reports, a Web-based dashboard, and a mobile app for smartphone users. Additional digital health platform details have been described elsewhere [13,18].

### Participant Enrollment

The clinical study enrolled adult and pediatric asthma patients (N=495) in parallel arms from specialty and primary care clinics at 2 sites in the Dignity Health system. Clinic staff enrolled eligible participants with the following inclusion criteria: over the age of 5 years, a provider diagnosis of asthma, presence of a SABA prescription at study inception, Spanish or English fluency, and absence of significant comorbidity such as chronic obstructive pulmonary disease. Further study detail is described elsewhere [13].

### Study Design

Participants were randomly assigned to either the intervention or control group and matched on sex, age, insurance type (public vs private), and baseline asthma control status (as defined by an Asthma Control Test [ACT] score ≤19 indicating a lack of asthma control) [29]. All participants received at least one sensor to monitor their medication over a 12-month period. If a participant had a smartphone, the study coordinator assisted them in downloading the app and conducting a first sync with the sensor. If a participant did not have a smartphone, they were provided a hub base station. This study used the earliest version of the sensor, which had a 15-day battery life, and participants were instructed to charge the sensor at regular intervals. Intervention group participants (n=250) received full access to the digital health platform described previously for 12 months. Physicians from the clinics could monitor the status of these intervention patients in real time and receive notifications about SABA overuse through Web-based dashboards. The Dignity Health Institutional Review Board reviewed and approved this study and survey (Trial Registration: ClinicalTrials.gov NCT01509183).

### Survey Data Collection

Surveys were administered electronically to participants in the intervention group who were aged 12 years or older at study completion at 12 months (N=207). The exit survey assessed asthma control with the ACT [29]. The study coordinator sent participants an email invitation to complete the survey electronically, and the coordinator made a single follow-up attempt with participants who did not respond within 1 week. Participants were given the option to take a paper version of the survey and return it through the mail. The participants did not receive any incentive to complete the exit survey.

The survey consisted of open- and closed-ended questions, which evaluated satisfaction with the reports and information, satisfaction with the sensor device, learnings as a result of platform use, identified triggers, quality of communications with health care providers, suggestions for improving the reports and sensor, and interest in continued use of the platform. Detailed survey questions are included in Multimedia Appendix 1.

### Data Analysis

Closed-ended questions were analyzed by determining the percentage of respondents who selected each of the possible responses. Thematic analysis of the open-ended responses in the survey followed the structured approach described by Braun and Clarke [30], where primary themes and subthemes were identified and coded according to specific usability and value topic areas. Two reviewers (KH and MB) independently assessed the open-ended questions, met to address discrepancies, and agreed on primary themes and subthemes. We report the number and percentage of participants who reported the primary themes and subthemes.

We assessed participant engagement with the platform by evaluating the mean email open rate, number of push notifications received, and number of participant sign-ins to the dashboard or app. We used a logistic regression model to evaluate if there were differences between those participants who took the exit survey compared with those who did not across the following variables: age (12-17 years vs 18 years and over), sex, insurance type (public vs private), device type (smartphone vs hub), asthma control at intake (controlled vs uncontrolled), syncing duration (time between the first and last sync), and syncing frequency (number of syncs between the first and last sync). Using a logistic regression modeling approach, we also evaluated whether these variables were associated with participants’ responses to the closed-ended questions on device and report satisfaction, usefulness, and interest in continued use of the platform. Responses were grouped based on binary categories, for example, satisfied versus not satisfied, useful versus not useful.

## Results

### Participants

Of the intervention group participants (N=250), 207 met the requirement of being aged 12 years or older at study completion to receive the exit survey. A total of 89 participants completed the exit survey (Table 1), which represented a 42.9% response rate (89/207). Among the participants who completed the survey (N=89), the average and median ages were 42.7 and 45 years, respectively; 85% (76/89) of participants were aged older than 18 years, 15% (13/89) were aged between 12 and 17 years, and 64% (57/89) of participants were female. Participants used a variety of device types to access the digital platform, including smartphones (49%, 44/89; 27%, 24/89 iOS and 23%, 20/89Android) and hub base stations for those without smartphones (51%, 45/89). In addition, 74% (66/89) of participants had private insurance and 26% (23/89) had public insurance. At the start of the study, 30% (27/89) of participants were well controlled, 61% (54/89) were not well controlled, and 9% (8/89) had an unknown control status.

Among the 207 intervention group participants, the logistic regression model identified that participants who used smartphones (*P*=.02), had a longer syncing duration (*P*<.001), and higher syncing frequency (*P*<.001) were more likely to take the exit survey (Table 2). There were no other significant differences across age, sex, asthma control, or insurance type between those participants who took the survey and those who did not.

Among the participants who completed the survey (N=89), on average, they synced their sensor for 361 days over the course of the study period (minimum: 264 days and maximum: 365 days). Participants synced their sensor 2149 times on average during the study or an average of 6 times per day. Participants also had a mean email open rate of 78%, received 35 push notifications, and had a mean 60 sign-ins to the patient dashboard, which represents more than 1 sign-in per week. The following results pertain to the 89 participants who completed the survey.

### Satisfaction With Inhaler Sensor Device

**Figure 2:**
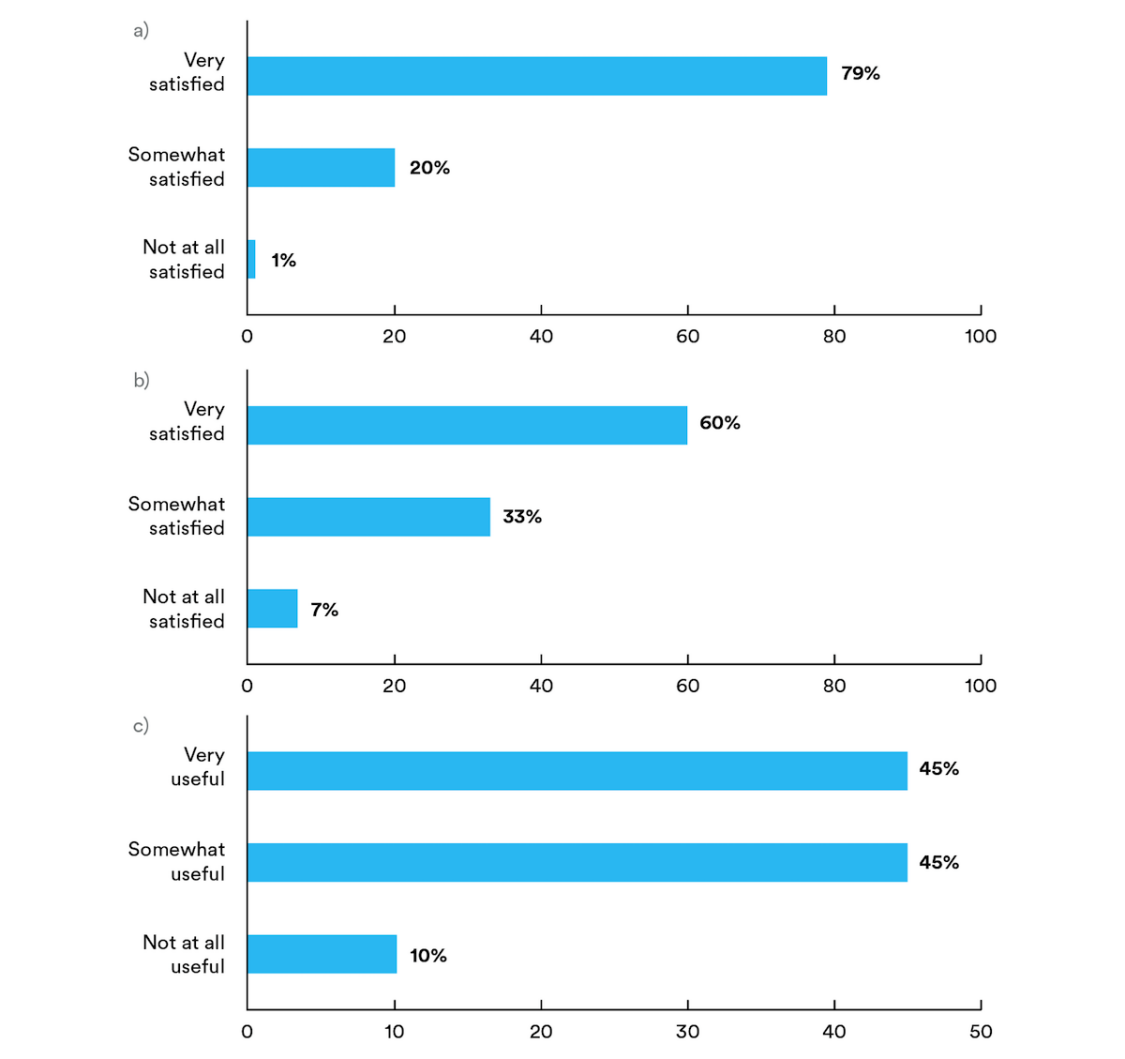
Participants’ responses to closed-ended questions: a) “How satisfied were you with the inhaler device?” b) “Overall, how satisfied were you with the reports?” and c) “How useful were the reports in helping you learn more about your asthma?”.

Participants reported their degree of satisfaction with the inhaler sensor device, with 79% (70/89) of respondents reporting “very satisfied,” 20% (18/89) reporting “somewhat satisfied,” and 1% (1/89) reporting “not at all satisfied” (Figure 2). There were no significant differences in responses to this question across participants’ device type, age, sex, insurance, asthma control at intake, or syncing history (see Multimedia Appendix 2). A total of 37 participants (40%, 37/89) stated the inhaler device was “easy,” including 3 subthemes of “easy to use” (32%, 29/89), “easy to maintain” (6%, 6/89), and “easy to understand” (2%, 2/89). Moreover, 23 participants (26%, 23/89) described the size of the device as “small” and “compact” (Table 3). Of the subthemes relating to the size of the device, 10 participants (11%, 10/89) specified that it did not “obstruct” or “interfere” with their use of their inhaler and “was not bulky.” Furthermore, 17 participants (19%, 17/89) described the sensor’s convenience, with the 2 subthemes of the device being “portable” and functioning well.

The majority of the participants (56%, 50/89) made no recommendations for sensor improvements. Of the participants who reported specific ideas for improvements (44%, 39/89), the most frequent response was for a longer battery life (20%, 18/89). Participants also reported wanting a smaller size device (13%, 12/89). The remaining participants reported wanting a more secure fit (4%, 4/89) or recommended additional features such as adding a dose counter (6%, 5/89).

**Table 1:** Characteristics of participants who responded to the exit survey (N=89).

Characteristics of participants		n (%)
**Device**		
	Smartphone	44 (49)
	Hub	45 (51)
**Asthma control**		
	Well controlled	27 (30)
	Not well controlled	54 (61)
	Unknown	8 (9)
**Age (years)**		
	12-17	13 (15)
	≥18	76 (85)
**Sex**		
	Female	57 (64)
	Male	32 (36)
**Insurance**		
	Private	66 (74)
	Public	23 (26)

**Table 2:** Demographic and individual predictors of whether participants completed survey or not (N=207).

Characteristics of participants	Predictors of survey completion (N=207)	
	Estimate (SE)	*P* value
Device type (smartphone)	1.154 (0.508)	.02
Age <18 years	0.157 (0.640)	.81
Syncing duration	0.063 (0.016)	<.001
Syncing frequency	0.008 (0.002)	<.001
Sex (male)	0.019 (0.438)	.97
Insurance (public)	−0.118 (0.481)	.81
Initial uncontrolled asthma	−0.686 (0.854)	.42
Initial well-controlled asthma	−0.471 (0.868)	.59

**Table 3:** Primary themes, subthemes, and selected excerpts from open-ended responses about participants’ experience with the inhaler sensor device.

Primary and subthemes		Representative quotes
**Easy**		
	Easy to use	“It is easy to use.” “It was easy to operate.”
	Easy to understand	“Instructions for use are pretty straight forward.” “Easy to understand.”
	Easy to maintain	“Easy to attach to my inhaler.” “Easy to use [and] care for.”
**Size**		
	Small	“Small and compact.” “Small size.”
	Unobtrusive	“Didn’t obstruct anything.” “Didn't get in the way.” “Not big or bulky which I liked.”
**Convenience**		
	Portable	“Easy to keep with you. Easy to carry.” “Fits in purse.”
	Functioned well	“Worked great.” “I liked that it worked wherever I went.”

### Perception of the Reports and Information

Participants reported on their satisfaction and perceptions of the reports’ usability and value in supporting their asthma management. A total of 60% (54/89) of the participants reported that they were “very satisfied” with the reports, 33% (29/89) were “somewhat satisfied,” and 7% (6/89) were “not at all satisfied” (Figure 2). There were no significant differences in responses to this question across participants’ device type, age, sex, insurance, asthma control at intake, or syncing history (see Multimedia Appendix 3).

Moreover, 33 (37%, 33/89) of participants reported liking the content of the reports, including informative (9%, 8/89), actionable (6%, 5/89), and easy to understand (8%, 7/89; Table 4). They also reported that they liked the report display (10%, 9/89) and the frequency (4%, 4/89). In addition, 23 participants (26%, 23/89) shared that the reports provided new information that increased their awareness of their asthma management. Some participants did not see significant value in the reports, responding that they did not view the reports (9%, 8/89), found the reports to be only “generally helpful” (4%, 4/89), did not receive the reports (3%, 3/89), or did not have any feedback to share (9%, 8/89).

### Perceived Utility and Participant Learnings

Participants responded the information was “very useful” (45%, 40/89), “somewhat useful” (45%, 40/89), or “not at all useful” (10%, 9/89) in learning about their asthma (Figure 2). There were no significant differences in responses to this question across participants’ device type, age, sex, insurance, asthma control at intake, or syncing history (see Multimedia Appendix 4).

The participants reported several learnings from using the digital platform (Table 5). A primary theme focused on understanding, identifying, and managing asthma triggers (23%, 21/89). A total of 53 participants (60%, 53/89) reported identifying up to 7 new triggers. Weather, allergies, and exercise were the most commonly identified triggers, documented across 79% (42/53) of the participants who identified new triggers.

A second primary theme focused on improved self-awareness. A total of 19 participants (21%, 19/89) specifically reported learning from monitoring the timing, location, and frequency of their medication use. Moreover, 10 participants (11%, 10/89) described learning strategies for managing their asthma such as taking their controller medications and avoiding specific triggers. Furthermore, 6 participants (7%, 6/89) reported that they discovered they were not as in control of their asthma as they had believed or they confirmed that their treatment plan helped them stay controlled. In addition, 5 participants (6%, 5/89) learned that they are accountable for their self-management and have the capacity to control their asthma. Participants could opt to discuss their data with their providers; 46% (41/89) of the participants reported that they had conversations with their providers about their data.

Some participants reported not learning anything from the reports: 16 participants (18%, 16/89) reported “none,” and 3 participants (3%, 3/89) reported not learning anything because they were already well controlled.

**Table 4:** Primary themes, subthemes, and selected excerpts from open-ended responses about participants’ experience with the data reports.

Primary and subthemes		Representative quotes
**Content of reports**		
	Informative	“Very informative.” “They contained relevant information.”
	Actionable	“Tells you about asthma so I can control it.” “Indicate to me where and when I needed to improve my surroundings to limit asthma problems.”
	Easy to understand	“Easy to read and understand.” “Easy to read [and] interpret.”
	Display (visual)	“Being able to see the data was helpful.” “Seeing a visual.”
	Frequency	“Regular feedback.” “Timely”
**New information that increased awareness**		
	Triggers	“Helped me try to focus on what my triggers are.” “Kept up to date, let me know weather and pollen count.”
	Timing and location of medication use	“I could see how often I was using the medication as well as the location.” “GPS [Global Positioning Systems] locations of incidents is nice.”
	Control	“Tell me how well my asthma was controlled or not controlled.” “Helps to show tracking of asthma control.”
	Frequency of medication use	“Knowing the actual number of time I needed meds.” “Kept me conscious of regular use of meds.”
	General understanding	“Told me how I was managing.” “They just allowed me to see the general picture about my condition.”

Participants were asked to provide suggestions for improving the reports or platform. A total of 59 participants (66%, 59/89) reported no improvements were needed, and 11 participants (12%, 11/89) responded with “not sure” or “no comment.” The remaining responses (21%, 19/89) offered specific suggestions including more educational content on how to improve asthma, information about how to subsidize medication costs, improvements in the app login process, additional customization options for how to receive reports, changing the cadence of the reports, and extending the sensor battery life.

The final question of the survey asked participants whether they would like to continue using the digital intervention: 72% (64/89) of the participants reported that they were interested in continuing to use the sensor and platform beyond the study. There were no significant differences among those participants who were interested in continuing or those who were not based on a participant’s device type, age, gender, insurance type, syncing history, or asthma control at intake (see Multimedia Appendix 5).

**Table 5:** Primary themes and subthemes of participant learnings as a result of the digital intervention.

Primary and subthemes		Representative quotes
**Triggers**		
	Specific triggers	“Worsens a lot with exercise.” “Stress can bring on symptoms.”
	To be aware of triggers	“Should pay more attention to what triggers attacks.” “I pay closer attention to my triggers.”
	Value of being aware of triggers	“Discovering the triggers of your asthma can better help you control it.” “By being aware of the pollen count assist in what activities to do when.”
**Medication use**		
	General awareness	“It made me much more aware of the use of my inhaler.” “How often I was using my inhaler.”
	Timing and location	“Learned that my asthma bothers me more at night and windy areas.” “I learned a lot about when and where my attacks were at.”
**Asthma control**		
	New insights	“I learned how well my asthma was controlled through those reports.” “That it wasn't as controlled as I thought.”
	Confirmation of control status	“I confirmed that my asthma is well-controlled with my current medication.” “It confirmed the combination of avoidance of known triggers and the medication keeps it well managed.”
Management strategies		“To make sure to take the preventative every time.” “How to stay away from what triggers my asthma.”
Management beliefs		“That I can control it better if I try.” “The program makes you accountable for taking the steps to control the asthma.”
Ways to communicate with doctor		“I was never sure how often I used my rescue inhaler. When my doctor asked I could not give him an accurate response and now I can.” “I learned that I need to talk to my doctor about my asthma during [and] post workouts.”

## Discussion

### Summary of Findings

Participants were satisfied with the digital health intervention for managing their asthma and perceived value in using the technology to support their self-management. Participants reported improving their awareness, learning about trends in their medication use, identifying new triggers, and objectively monitoring how well their asthma is controlled, which they could discuss with their doctor. Participants shared helpful feedback regarding areas for improvement including interest in longer battery life and smaller size of the device.

We saw no significant differences in the degree of satisfaction and perceived usability across participants regardless of participant age, gender, insurance type, asthma control, technology adoption, and syncing duration. However, we did observe that those with a smartphone and a longer record of syncing were more likely to take the survey in the first place, which could have biased the sample toward more engaged, more technologically savvy participants. Despite this potential bias, these results add to the limited evidence exploring patient perspectives on digital health tools across diverse participants and shed light on the perceived benefits of the tools as well as opportunities for improvement.

### Mechanisms for Supporting Self-Management

These findings align with and supplement the existing evidence [19,24,31], in particular adding to the literature by demonstrating participants’ perceptions among a larger cohort in a real-world setting over a prolonged period of time. We explore 4 learnings that emerged from the participants’ responses.

First, participants frequently emphasized ease of use for the inhaler sensor in terms of deployment, comprehension, maintenance, size, and convenience. Similarly, in a small study of adolescent asthma participants, patients cited the importance of the device not drawing any attention, being small in size, and being easily portable [28]. Furthermore, a review of previous digital health intervention studies suggested that ease of use and adaptation to an individual’s personal lifestyle are essential factors for engagement and persistent use [32]. These findings align with the concept of minimally disruptive medicine, which aims to find ways to reduce patient treatment burden [33]. To support this approach, digital tools enable passive data collection of medication use, eliminating the burden of manual tracking. By providing objective documentation of medication use, trends, and specific information about potential triggers, the self-monitoring process can become more efficient and actionable.

Second, participants in this study and others have reported that having objective data leads to increased self-awareness. This finding aligns with the self-regulation model for chronic disease and the more refined version for asthma management [24,34], in which a patient goes through a process of self-monitoring and assessing feedback to inform behavior. Participants reported gaining insights from monitoring medication use, especially the timing and location of medication use events, to identify patterns, times of worsening, and potential triggers. Almost one-quarter of patients valued learning about their asthma triggers including specific environmental conditions, such as temperature, humidity, and air quality, through the digital tool to help inform their management.

Improved confidence in one’s ability to self-manage, as was seen in some of the open-ended responses, is promising, but further investigation is needed to evaluate the intervention’s impact on self-efficacy. The survey design did not include specific questions to assess self-efficacy; therefore, we are unable to determine if the intervention had an impact on self-efficacy. Future studies evaluating digital interventions for asthma should thoroughly evaluate self-efficacy using validated self-efficacy measures and surveys, such as the mini-Asthma Quality of Life Questionnaire [35], Self-efficacy and Situational Barrier Survey Questionnaire (KASE-AQ) [36], and Mobile App Rating Scale [37].

Third, this study found that participants value having information they can share with their clinical providers. Almost half of all participants had spoken with their clinical provider about the data collected. A smaller subset reported using the data to request medication adjustments, therefore taking more responsibility for their management. These findings are supported in a study that found that 70% of patients wished to share their data with health teams [31].

Digital health tools can support the patient-provider relationship through shared decision making [38,39]. By enabling the sharing of data and the ability of patients to discuss specific issues as they arise, patients and providers can work collaboratively to adjust their management plan and achieve asthma control. A recent study that surveyed clinical providers about digital health tools, such as sensors, documented similar perceived benefits. Clinicians reported that objective data from sensors have the potential to enable patients to see trends and patterns in their medication use, increase accountability and confidence in their management, and support discussion with clinical providers [40].

Fourth, this study identified areas for improvement that can inform digital health tool development in the future. Participants reported wanting a longer battery life for the sensors. This feedback helped inform the development of the latest version of the sensors, which use Bluetooth low energy technology, do not require charging, and have a battery life of 12-18 months. In addition, some participants reported an interest in a smaller device, which resulted in the development of a sensor with 30% reduced volume. Participants also reported desiring more personalization and customization in the notifications and reminders and requested multiple methods of communication so that they can select the method that works best for them at the right time. Ongoing work is underway to add personalization, customization, and new communication options. There is growing evidence in this study and others [24,31] indicating how much patients value personalization and customization; however, more research is needed to determine best practices for identifying customizable features that will enhance patient satisfaction and engagement.

### Limitations

First, although the participants’ cohort was fairly diverse in age, insurance type, technology use, gender, and asthma control status, additional studies are needed to evaluate preferences in larger samples of different types of patients across race, pediatric versus adult, geographic diversity, and health literacy levels. For example, this survey was only administered to participants aged 12 years and older and therefore did not capture the young pediatric patient or caregiver perspective. Second, the response rate was 42.9%, and selection bias may have influenced the results. We did find that participants who used smartphones and those who remained in the program longer (defined by their sync duration) were more likely to take the survey in the first place. They may have experienced more value from a longer interaction with the intervention and reported as such on the survey. Future research should target individuals with early study attrition to ensure a more complete understanding of their perceptions of usability and satisfaction to enable scalability to all populations. Third, the infrastructure to capture more robust individual-level participant engagement data was limited, which prevented a more complete understanding of participant engagement and how this engagement may have influenced overall satisfaction and perception of utility. Fourth, the survey questions were not derived from validated measures and did not use standard Likert scales, which limited the generalizability of the results.

### Conclusions

This study offers new insights about patient satisfaction, preferences, and perceptions of a digital health intervention for respiratory disease among a larger, fairly diverse cohort in a real-world setting after a prolonged period of use. Participants reported satisfaction with the sensor device and reports and particularly valued the ability to integrate the device into everyday life, the provision of accessible and meaningful information to improve self-awareness, and the enhancement of communication with health care providers. As the use of digital tools in health care expands, it will be critical to learn from patient preferences and experiences to ensure that the tools fit well into their daily life and support their self-management.

## Appendix

Multimedia Appendix 1 Survey questions.

Multimedia Appendix 2 Participants’ responses to the closed-ended question, “How satisfied were you with the inhaler device?” by demographic and individual characteristics.

Multimedia Appendix 3 : Participants’ responses to the closed-ended question, “Overall, how satisfied were you with the reports?” by demographic and individual characteristics.

Multimedia Appendix 4 Participants’ responses to the closed-ended question, “How useful were the reports in helping you learn more about your asthma?” by demographic and individual characteristics.

Multimedia Appendix 5 Participants’ responses to the closed-ended question, “How interested would you be in continuing to track your asthma medication use with this program?” by demographic and individual characteristics.

## Abbreviations

ACT: Asthma Control Test
KASE-AQ: The Knowledge, Attitude, and Self-Efficacy Asthma Questionnaire
SABA: short-acting beta-agonist

## References

[1] (2017). Centers for Disease Control.

[2] Stanford RH, Gilsenan AW, Ziemiecki R, Zhou X, Lincourt WR, Ortega H (2010). Predictors of uncontrolled asthma in adult and pediatric patients: analysis of the Asthma Control Characteristics and Prevalence Survey Studies (ACCESS). J Asthma.

[3] Barnett SB, Nurmagambetov TA (2011). Costs of asthma in the United States: 2002-2007. J Allergy Clin Immunol.

[4] Bårnes CB, Ulrik CS (2015). Asthma and adherence to inhaled corticosteroids: current status and future perspectives. Respir Care.

[5] Bender BG, Pedan A, Varasteh LT (2006). Adherence and persistence with fluticasone propionate/salmeterol combination therapy. J Allergy Clin Immunol.

[6] Williams LK, Peterson EL, Wells K, Campbell J, Wang M, Chowdhry VK, Walsh M, Enberg R, Lanfear DE, Pladevall M (2010). A cluster-randomized trial to provide clinicians inhaled corticosteroid adherence information for their patients with asthma. J Allergy Clin Immunol.

[7] Wu AC, Butler MG, Li L, Fung V, Kharbanda EO, Larkin EK, Vollmer WM, Miroshnik I, Davis RL, Lieu TA, Soumerai SB (2015). Primary adherence to controller medications for asthma is poor. Ann Am Thorac Soc.

[8] Huckvale K, Morrison C, Ouyang J, Ghaghda A, Car J (2015). The evolution of mobile apps for asthma: an updated systematic assessment of content and tools. BMC Med.

[9] Chan AH, Stewart AW, Harrison J, Camargo Jr CA, Black PN, Mitchell EA (2015). The effect of an electronic monitoring device with audiovisual reminder function on adherence to inhaled corticosteroids and school attendance in children with asthma: a randomised controlled trial. Lancet Respir Med.

[10] Charles T, Quinn D, Weatherall M, Aldington S, Beasley R, Holt S (2007). An audiovisual reminder function improves adherence with inhaled corticosteroid therapy in asthma. J Allergy Clin Immunol.

[11] Foster JM, Usherwood T, Smith L, Sawyer SM, Xuan W, Rand CS, Reddel HK (2014). Inhaler reminders improve adherence with controller treatment in primary care patients with asthma. J Allergy Clin Immunol.

[12] Krishna S, Francisco BD, Balas EA, König P, Graff GR, Madsen RW, Randomized trial (2003). Internet-enabled interactive multimedia asthma education program: a randomized trial. Pediatrics.

[13] Merchant RK, Inamdar R, Quade RC (2016). Effectiveness of population health management using the propeller health asthma platform: a randomized clinical trial. J Allergy Clin Immunol Pract.

[14] Petrie KJ, Perry K, Broadbent E, Weinman J (2012). A text message programme designed to modify patients' illness and treatment beliefs improves self-reported adherence to asthma preventer medication. Br J Health Psychol.

[15] Prabhakaran L, Chee WY, Chua KC, Abisheganaden J, Wong WM (2010). The use of text messaging to improve asthma control: a pilot study using the mobile phone short messaging service (SMS). J Telemed Telecare.

[16] Strandbygaard U, Thomsen SF, Backer V (2010). A daily SMS reminder increases adherence to asthma treatment: a three-month follow-up study. Respir Med.

[17] van der Meer V, Bakker MJ, van den Hout WB, Rabe KF, Sterk PJ, Kievit J, Assendelft WJ, Sont JK, SMASHING (Self-Management in Asthma Supported by Hospitals, ICT, Nurses and General Practitioners) Study Group (2009). Internet-based self-management plus education compared with usual care in asthma: a randomized trial. Ann Intern Med.

[18] Van Sickle D, Magzamen S, Truelove S, Morrison T (2013). Remote monitoring of inhaled bronchodilator use and weekly feedback about asthma management: an open-group, short-term pilot study of the impact on asthma control. PLoS One.

[19] Cook KA, Modena BD, Simon RA (2016). Improvement in Asthma Control Using a Minimally Burdensome and Proactive Smartphone Application. J Allergy Clin Immunol Pract.

[20] Miller L, Schüz B, Walters J, Walters EH (2017). Mobile technology interventions for asthma self-management: systematic review and meta-analysis. JMIR Mhealth Uhealth.

[21] Mosnaim G, Li H, Martin M, Richardson D, Belice PJ, Avery E, Silberstein A, Leigh J, Kenyon R, Jones S, Bender B, Powell LH (2015). A tailored mobile health intervention to improve adherence and asthma control in minority adolescents. J Allergy Clin Immunol Pract.

[22] Licskai C, Sands TW, Ferrone M (2013). Development and pilot testing of a mobile health solution for asthma self-management: asthma action plan smartphone application pilot study. Can Respir J.

[23] Burbank AJ, Lewis SD, Hewes M, Schellhase DE, Rettiganti M, Hall-Barrow J, Bylander LA, Brown RH, Perry TT (2015). Mobile-based asthma action plans for adolescents. J Asthma.

[24] Sage A, Roberts C, Geryk L, Sleath B, Tate D, Carpenter D (2017). A self-regulation theory-based asthma management mobile app for adolescents: a usability assessment. JMIR Hum Factors.

[25] Sawesi S, Rashrash M, Phalakornkule K, Carpenter JS, Jones JF (2016). The impact of information technology on patient engagement and health behavior change: a systematic review of the literature. JMIR Med Inform.

[26] Morrison D, Wyke S, Agur K, Cameron EJ, Docking RI, Mackenzie AM, McConnachie A, Raghuvir V, Thomson NC, Mair FS (2014). Digital asthma self-management interventions: a systematic review. J Med Internet Res.

[27] Kenyon CC, Chang J, Wynter SA, Fowler JC, Long J, Bryant-Stephens TC (2016). Electronic adherence monitoring in a high-utilizing pediatric asthma cohort: a feasibility study. JMIR Res Protoc.

[28] Howard S, Lang A, Sharples S, Shaw D (2017). See I told you I was taking it! - Attitudes of adolescents with asthma towards a device monitoring their inhaler use: implications for future design. Appl Ergon.

[29] Nathan RA, Sorkness CA, Kosinski M, Schatz M, Li JT, Marcus P, Murray JJ, Pendergraft TB (2004). Development of the asthma control test: a survey for assessing asthma control. J Allergy Clin Immunol.

[30] Braun V, Clarke V (2014). What can “thematic analysis” offer health and wellbeing researchers?. Int J Qual Stud Health Well-being.

[31] Simpson AJ, Honkoop PJ, Kennington E, Snoeck-Stroband JB, Smith I, East J, Coleman C, Caress A, Chung KF, Sont JK, Usmani O, Fowler SJ (2017). Perspectives of patients and healthcare professionals on mHealth for asthma self-management. Eur Respir J.

[32] O'Connor S, Hanlon P, O'Donnell CA, Garcia S, Glanville J, Mair FS (2016). Understanding factors affecting patient and public engagement and recruitment to digital health interventions: a systematic review of qualitative studies. BMC Med Inform Decis Mak.

[33] May C, Montori VM, Mair FS (2009). We need minimally disruptive medicine. BMJ.

[34] Clark NM, Gong M, Kaciroti N (2014). A model of self-regulation for control of chronic disease. Health Educ Behav.

[35] Juniper EF, Guyatt GH, Cox FM, Ferrie PJ, King DR (1999). Development and validation of the Mini Asthma Quality of Life Questionnaire. Eur Respir J.

[36] Wigal JK, Stout C, Brandon M, Winder JA, McConnaughy K, Creer TL, Kotses H (1993). The Knowledge, Attitude, and Self-Efficacy Asthma Questionnaire. Chest.

[37] Stoyanov SR, Hides L, Kavanagh DJ, Zelenko O, Tjondronegoro D, Mani M (2015). Mobile app rating scale: a new tool for assessing the quality of health mobile apps. JMIR Mhealth Uhealth.

[38] Charles C, Whelan T, Gafni A (1999). What do we mean by partnership in making decisions about treatment?. BMJ.

[39] Tapp H, Shade L, Mahabaleshwarkar R, Taylor YJ, Ludden T, Dulin MF (2017). Results from a pragmatic prospective cohort study: shared decision making improves outcomes for children with asthma. J Asthma.

[40] Howard S, Lang A, Sharples S, Shaw D (2016). What are the pros and cons of electronically monitoring inhaler use in asthma? A multistakeholder perspective. BMJ Open Respir Res.

