# Supplementary material for: Digital Health Intervention for Asthma: Patient-Reported Value and Usability
Source: JMIR Mhealth Uhealth. 2018 Jun 4;6(6):e133. doi: 10.2196/mhealth.7362 (PMC6006012; doi:10.2196/mhealth.7362)
Supplement: Multimedia Appendix 1 [file mhealth_v6i6e133_app1.pdf]

Multimedia Appendix 1. Survey Questions

In the past 4 weeks, how much of the time did your asthma keep you from getting as much done at work, school, or home? (ACT question 1)

- All of the time
- Most of the time
- Some of the time
- A little of the time
- None of the time

During the past 4 weeks, how often have you had shortness of breath? (ACT question 2)

- More than once a day
- Once a day
- 3 to 6 times a week
- Once or twice a week
- Not at all

During the past 4 weeks, how often did your asthma symptoms (wheezing, coughing, shortness of breath, chest tightness or pain) wake you up at night or earlier than usual in the morning? (ACT question 3)

- 4 or more nights a week
- 2 or 3 nights a week
- Once a week
- Once or twice
- Not at all

During the past 4 weeks, how often have you used your rescue inhaler or nebulizer medication (such as albuterol)? (ACT question 4)

- 3 or more times per day
- 1 or two times per day
- 2 or 3 times per week
- Once a week or less
- Not at all

How would you rate your asthma control during the past 4 weeks? (ACT question 5)

- Not controlled at all
- Poorly controlled
- Somewhat controlled
- Well controlled
- Completely controlled

How useful were the reports in helping you learn more about your asthma?

- Very useful
- Somewhat useful
- Not at all useful

What did you like about the reports?

How could we improve the reports to better meet your needs or interests?

Overall, how satisfied were you with the reports?
- Very satisfied
- Somewhat satisfied
- Not at all satisfied

How satisfied were you with the inhaler device?
- Very satisfied
- Somewhat satisfied
- Not at all satisfied

What did you like about the design or operation of the inhaler device?

How could we improve usability or design of the inhaler device to better meet your needs?

Please provide any other comments or suggestions you have about the inhaler device.

Did you ever talk with your healthcare provider about your asthma as a result of information you received from this project?
- Yes. My healthcare provider initiated the conversation.
- Yes. I initiated the conversation.
- No

Please describe what you learned about your asthma by participating in this project.

Please describe any triggers or exposures for your asthma that you identified by participating in this project.

Are you interested in continuing to track your asthma medication use with this program?
