# Supplementary material for: Digital Health Intervention for Asthma: Patient-Reported Value and Usability
Source: JMIR Mhealth Uhealth. 2018 Jun 4;6(6):e133. doi: 10.2196/mhealth.7362 (PMC6006012; doi:10.2196/mhealth.7362)
Supplement: Multimedia Appendix 2 [file mhealth_v6i6e133_app2.pdf]

Multimedia Appendix 2. Participants' responses to the closed-ended question, "How satisfied were you with the inhaler device?," by demographic and individual characteristics

	Estimate	Std. Error	P-value
Device Type (Smartphone)	-70.850	39970	>0.99
Age < 18	8.964	63720	>0.99
Syncing Duration	-0.708	6697	>0.99
Syncing Frequency	0.249	133	>0.99
Sex (Male)	-75.460	45720	>0.99
Insurance (Public)	-15.710	55980	>0.99
Initial Uncontrolled Asthma	34.290	149600	>0.99
Initial Well Controlled Asthma	-33.700	151700	>0.99
