# Supplementary material for: Digital Health Intervention for Asthma: Patient-Reported Value and Usability
Source: JMIR Mhealth Uhealth. 2018 Jun 4;6(6):e133. doi: 10.2196/mhealth.7362 (PMC6006012; doi:10.2196/mhealth.7362)
Supplement: Multimedia Appendix 3 [file mhealth_v6i6e133_app3.pdf]

Multimedia Appendix 3. Participants' responses to the closed-ended question, "Overall, how satisfied were you with the reports?," by demographic and individual characteristics

	Estimate	Std. Error	P-value
Device Type (Smartphone)	2.306	1.275	0.07
Age < 18	17.890	4273	>0.99
Syncing Duration	-0.872	225	>0.99
Syncing Frequency	0.006	0.005	0.28
Sex (Male)	1.212	1.215	0.32
Insurance (Public)	-0.904	0.998	0.37
Initial Uncontrolled Asthma	-15.180	5069	>0.99
Initial Well Controlled Asthma	-15.420	5069	>0.99
