# Supplementary material for: Digital Health Intervention for Asthma: Patient-Reported Value and Usability
Source: JMIR Mhealth Uhealth. 2018 Jun 4;6(6):e133. doi: 10.2196/mhealth.7362 (PMC6006012; doi:10.2196/mhealth.7362)
Supplement: Multimedia Appendix 4 [file mhealth_v6i6e133_app4.pdf]

Multimedia Appendix 4. Participants' responses to the closed-ended question, "How useful were the reports in helping you learn more about your asthma?," by demographic and individual characteristics

	Estimate	Std. Error	P-value
Device Type (Smartphone)	1.666	0.945	0.08
Age < 18	18.510	4414.000	>0.99
Syncing Duration	-0.947	224.200	>0.99
Syncing Frequency	0.002	0.004	0.60
Sex (Male)	0.721	0.896	0.42
Insurance (Public)	-0.560	0.817	0.49
Initial Uncontrolled Asthma	-16.440	5252.000	>0.99
Initial Well Controlled Asthma	-16.650	5252.000	>0.99
