# Supplementary material for: Digital Health Intervention for Asthma: Patient-Reported Value and Usability
Source: JMIR Mhealth Uhealth. 2018 Jun 4;6(6):e133. doi: 10.2196/mhealth.7362 (PMC6006012; doi:10.2196/mhealth.7362)
Supplement: Multimedia Appendix 5 [file mhealth_v6i6e133_app5.pdf]

Multimedia Appendix 5. Participants' responses to the closed-ended question, "How interested would you be in continuing to track your asthma medication use with this program?," by demographic and individual characteristics

	Estimate	Std. Error	P-value
Device Type (Smartphone)	0.204585	0.631053	0.75
Age < 18	0.233965	0.874351	0.79
Syncing Duration	0.080496	0.044419	0.07
Syncing Frequency	0.005054	0.002959	0.09
Sex (Male)	1.198352	0.665589	0.07
Insurance (Public)	-0.076924	0.670103	0.91
Initial Uncontrolled Asthma	-1.594618	1.809582	0.38
Initial Well Controlled Asthma	-3.023383	1.797173	0.09
